# Acute vascular response to cediranib treatment in human non-small-cell lung cancer xenografts with different tumour stromal architecture

**DOI:** 10.1016/j.lungcan.2015.08.009

**Published:** 2015-11

**Authors:** Yanyan Jiang, Danny Allen, Veerle Kersemans, Aoife M. Devery, Sivan M. Bokobza, Sean Smart, Anderson J. Ryan

**Affiliations:** CRUK & MRC Oxford Institute for Radiation Oncology, Department of Oncology, University of Oxford, Roosevelt Drive, Oxford OX3 7DQ, United Kingdom

**Keywords:** NSCLC, Cediranib, VEGF, Tumour vasculature, Blood perfusion, Hypoxia

## Abstract

**Objectives:**

Tumours can be categorised based on their stromal architecture into tumour vessel and stromal vessel phenotypes, and the phenotypes have been suggested to define tumour response to chronic treatment with a VEGFR2 antibody. However, it is unclear whether the vascular phenotypes of tumours associate with acute vascular response to VEGFR tyrosine kinase inhibitors (TKI), or whether the early changes in vascular function are associated with subsequent changes in tumour size. This study was sought to address these questions by using xenograft models of human non-small cell lung cancer (NSCLC) representing stromal vessel phenotype (Calu-3) and tumour vessel phenotype (Calu-6), respectively.

**Methods:**

For dynamic contrast-enhanced magnetic resonance imaging (DCE-MRI), nude mice bearing established Calu-3 or Calu-6 xenografts were treated with a potent pan-VEGFR TKI, cediranib (6 mg/kg), at 0 h and 22 h. DCE-MRI was performed 2 h before the first dose and 2 h after the second dose of cediranib to examine acute changes in tumour vessel perfusion. Tumours were harvested for hypoxia detection by CA9 immunohistochemistry. For tumour growth study, mice carrying established Calu-3 or Calu-6 tumours were treated with cediranib once daily for 5 days.

**Results:**

Twenty-four hours after cediranib administration, the perfusion of Calu-3 tumours was markedly reduced, with a significant increase in hypoxia. In contrast, neither perfusion nor hypoxia was significantly affected in Calu-6 tumours. Tumour regressions were induced in Calu-3 xenografts, but not in Calu-6 xenografts, although there was a trend towards tumour growth inhibition after 5 days of cediranib treatment.

**Conclusion:**

These findings suggest that tumour stromal architecture may associate with acute tumour vascular response to VEGFR TKI, and this acute tumour vascular response may be a promising early predictive marker of response to VEGFR TKI in NSCLC.

## Highlights

• We studied cediranib, a VEGFR tyrosine kinase inhibitor in lung cancer xenografts.
• Gadolinium-enhanced DCE-MRI was used to study acute vascular responses.
• Acute vascular response was associated with tumour stromal architecture.
• Tumour growth inhibition by cediranib was linked to acute vascular response.
• Acute vascular changes are a potential predictive marker of response to cediranib.

## 1 Introduction

Despite recent advances in cancer treatment, the 5-year survival of non-small cell lung cancer (NSCLC) remains low. Angiogenesis is essential for tumour growth, invasion, and metastasis by supplying nutrients and oxygen [1], [2], and is correlated with poor prognosis of NSCLC [3], [4]. Signalling through tyrosine kinase (TK) receptors including vascular endothelial growth factor (VEGF) receptor (VEGFR), platelet-derived growth factor receptor (PDGFR), and fibroblast growth factor receptor (FGFR) plays a critical role in tumour angiogenesis [5], and consequently, inhibiting these receptors has emerged as a compelling approach for cancer treatment. Indeed, antiangiogenic therapy, particularly anti-VEGF/VEGFR therapy, has shown promise in treating NSCLC, alone or in combination with chemotherapy [6], [7], [8]. However, despite some benefits in the clinic, individual responses to anti-angiogenic agents are variable with many patients failing to benefit. Unfortunately, there are not yet any validated predictive biomarkers for patient selection for anti-angiogenic therapy.

As anti-angiogenic treatment-induced changes in tumour vascularity occur ahead of the reduction in tumour size, measurement of functional changes in tumour vessels may identify early response to anti-angiogenic treatment. Dynamic contrast-enhanced magnetic resonance imaging (DCE-MRI) is a non-invasive imaging modality that can detect changes in tumour perfusion, and it has been used to assess anti-vascular therapy response in both preclinical and clinical studies [9], [10], [11], [12], [13].

Based on stromal architecture, it has been proposed that human tumours can be categorised into two phenotypes: the tumour vessel phenotype in which blood vessels are distributed amongst tumour cells, and the stromal vessel phenotype in which blood vessels are embedded in stroma [14]. Importantly, these two phenotypes appear to define the tumour response to chronic inhibition of VEGF-signalling using the anti-VEGFR2 antibody, DC101 [14]. Compared to anti-VEGFR antibodies, VEGFR tyrosine kinase inhibitors (TKI) have broader pharmacology profiles and may inhibit additional kinases, thereby causing additional effects on tumour vasculature. However, it is unknown whether these vessel phenotypes associate with an acute pharmacodynamic vascular response to VEGFR TKI, or whether the early changes in vascular function associate with later changes in tumour size. To address these questions, we used Calu-3 and Calu-6 human NSCLC xenograft models to represent stromal vessel and tumour vessel phenotypes, respectively, and treated tumour-bearing mice with cediranib, a highly potent pan-VEGFR TKI with additional activity against c-Kit, PDGFR and FGFR [15]. Cediranib has shown anti-angiogenic and anti-tumour activity in multiple preclinical models of human cancer [15] and in clinical trials [16], [17], [18]. Here, we assessed changes in tumour perfusion and hypoxia after cediranib administration using DCE-MRI and immunohistochemistry, and compared the vascular functional changes with tumour growth inhibition by cediranib.

## 2 Materials and methods

### 2.1 Human NSCLC tumour tissues

Formalin-fixed, paraffin embedded NSCLC tumour samples (total *n *= 38; adenocarcinoma (*n *= 14), squamous cell carcinoma (*n *= 24)) were obtained from ProteoGenex, Inc. (Culver City, USA) with appropriate ethical approval and with informed consent.

### 2.2 Cell culture

Cell lines were obtained from the American Type Culture Collection and cultured in advanced DMEM/F12 medium supplemented with 5% FBS, 2 mM glutamax, and 50 μg/ml penicillin/streptomycin in a humidified atmosphere with 7.5% CO_2_. Cell line identity was confirmed by sequencing of highly polymorphic loci in the mitochondrial DNA [19].

### 2.3 Subcutaneous xenografts

All animal experiments were performed under a licence issued under the UK Animals (Scientific Procedures) Act of 1986. To establish xenografts, female BALB/C nude mice (6–8 weeks old, Harlan, Wolverhampton, UK) were anaesthetised with 2% isoflurane and 5 × 10^6^ tumour cells in 50% Matrigel (BD Biosciences) were subcutaneously injected at a single site on the back of the mouse. Mouse weights and tumour volumes were measured three times per week (volume = 1/2 × length × width × depth).

### 2.4 Cediranib treatment

When tumours reached approximately 150 mm^3^, mice were randomised into two groups. For DCE-MRI study, mice (*n *= 3/group) were dosed with either cediranib (AZD2171, Selleck Chemicals, Houston, USA) 6 mg/kg or vehicle 1% polysorbate by oral gavage at 0 h and 22 h. Tumours were imaged by DCE-MRI 2 h before the first treatment and 2 h after the second treatment of cediranib or vehicle. Animals were sacrificed immediately after the second DCE-MRI. For tumour growth study, mice (*n *= 3/group) were treated with either cediranib or vehicle 1% polysorbate once daily for 5 days. Tumour volumes were measured over the course of treatment.

### 2.5 DCE-MRI

MRI was performed in a 4.7T horizontal magnet (Agilent Technologies, Stockport, UK) using a 25 mm quadrature birdcage coil. Respiration-gated 3D gradient echo imaging (TE = 0.55 ms, TR = 1.1 ms, flip angle 5 degrees) covering a field of view of 54 × 27 × 27 mm at an isotropic resolution of 0.42 mm was used for the DCE. T1 was quantitatively mapped using variable flip angles nominally in the range of 0.5–8 degrees with RF transmission field inhomogeneities accounted for [20]. 30 μl of 0.5 M contrast agent gadodiamide (Gd, GE Healthcare) was infused via a tail vein cannula, and its uptake was monitored using 100 repetitions of the respiratory-gated scan.

The DCE-MRI signal was converted to gadolinium concentration as described [21]. The tumour region was manually segmented from an average image of the DCE-MRI time course using ITK-SNAP [22]. The initial area under the gadolinium uptake curve (IAUC) was calculated for the first 90 s on a voxel by voxel basis using in-house software written in Matlab as previously described [23], and the median for the tumour was determined [24].

### 2.6 Immunohistochemistry

Sections (4 μm) from paraffin-embedded tumours from xenografts or NSCLC tumour samples were deparaffinised followed by heat-induced epitope retrieval in citrate buffer (pH6.0). Sections were stained for CA9 (carbonic anhydrase 9) or CD31 using Dako EnVision™ - Dual link system-HRP (Dako) according to the manufacturer's instructions. The antibodies used for immunohistochemistry were: CA9 (mouse M75 monoclonal, AB1001, BioScience, 1:800) and CD31 (rabbit polyclonal, ab28364, Abcam, 1:50). Hematoxylin and eosin (H&E) and Masson's trichrome staining were performed for routine histological examination. Human NSCLC tumours were scored for stromal vessel and tumour vessel phenotypes based on CD31 and Masson's trichrome stains. The presence of intravascular erythrocytes identified functional vessels. Tumours showing strong predominance (≥90%) of functional vessels within desmoplastic stroma or within tumour tissue were defined as either stromal vessel or tumour vessel phenotypes, respectively. Where functional vessels consisted of >10% of each phenotype, the tumours were scored as a mixed vessel phenotype. Similarly, 12 NSCLC xenografts (PC9, H1975, H2087, H1395, COR-L105, A549, HCC827, Calu-6, Calu-3, H460, H3122, H1299) and an additional 31 xenografts or allografts from other types of tumours (HL-60, KARPAS-299, MOLM-13, MV4-11, C6, SK-N-MC, Colo-205, HCT-116, HT29, LoVo, LS-174T, RKO, SW620, MKN-1, MKN-45, Alexander, AsPC1, Mia-Paca2, RENCA, A375, A431, B16-F10, LNCaP, PC-3, MCF7, MDA-MB-231, C33A, HeLa, OVCAR-3, SKOV-3, NCI-H69) were assessed for vessel phenotypes.

### 2.7 Statistical analysis

Data were expressed as mean ± SEM. Statistical analysis was performed using Student's t-test by SPSS software. Statistical significance was defined as *P *< 0.05.

## 3 Results

### 3.1 NSCLC xenografts and patient samples display distinct tumour vessel and stromal vessel phenotypes

**Fig. 1:**
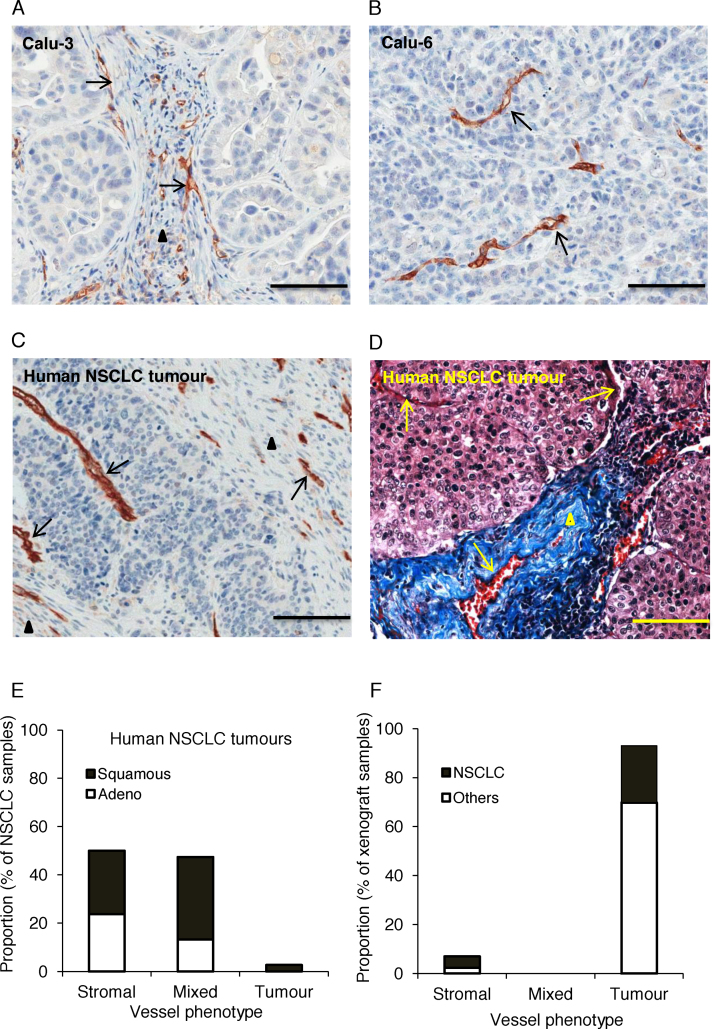
Distinct vessel phenotypes in NSCLC xenografts and human tumour samples. (A) CD31 immunohistochemistry staining in Calu-3 tumours. (B) CD31 immunohistochemistry staining in Calu-6 tumours. (C) CD31 immunohistochemistry staining in human NSCLC tumours showing a mixed vessel phenotype. (D) Masson's trichrome staining in human NSCLC tumours showing a mixed vessel phenotype. (E) Proportions of vessel phenotypes in human NSCLC tumours. (F) Proportions of vessel phenotypes in xenografts of NSCLC and other cancers grown in nude mice. Phenotypes were scored from CD31 and Masson's trichrome staining. Functional vessels were identified by the presence of intravascular erythrocytes. Arrows and arrow heads indicate vessels and stroma respectively. Scale bars = 100 μm.

Calu-3 and Calu-6 xenografts were stained for the endothelial cell marker CD31. Consistent with the previous report [14], Calu-3 tumours displayed the stromal vessel phenotype where the vessels are exclusively embedded within extensive desmoplastic stroma, which surrounded the tumour cells (Fig. 1A). In contrast, Calu-6 tumours showed exclusively a tumour vessel phenotype where vessels are distributed within the tumour tissue (Fig. 1B).

To investigate the relevance of the vessel phenotypes in human NSCLC, tumour samples were stained for CD31 (Fig. 1C) and with Masson’s trichrome (Fig. 1D). The mean (±SEM) area of tumour sections evaluated was 1.26 ± 0.08 cm^2^ and functional vessels were identified by the presence of intravascular erythrocytes. Among the human NSCLC tumour samples (*n *= 38), 19 exhibited a stromal vessel phenotype and 18 exhibited a mixed vessel phenotype, with only one sample showing exclusively a tumour vessel phenotype (Fig. 1E). Tumours with a mixed vessel phenotype were heterogeneous, with areas with vessels embedded in stroma adjacent to areas with vessels surrounded by tumour cells (Fig. 1C and D). The distribution between the vessel phenotypes was similar for both adenocarcinoma and squamous cell carcinoma (Fig. 1E). In contrast, only 2/12 of NSCLC xenograft models (HCC827, Calu-3) and 1/31 of other xenograft models (MKN-1) showed a stromal vessel phenotype with overall, the great majority of xenografts (40/43) showing a tumour vessel phenotype, and none showing a mixed vessel phenotype (Fig. 1F).

### 3.2 Gadolinium uptake in Calu-3 xenografts is decreased following acute cediranib treatment

**Fig. 2:**
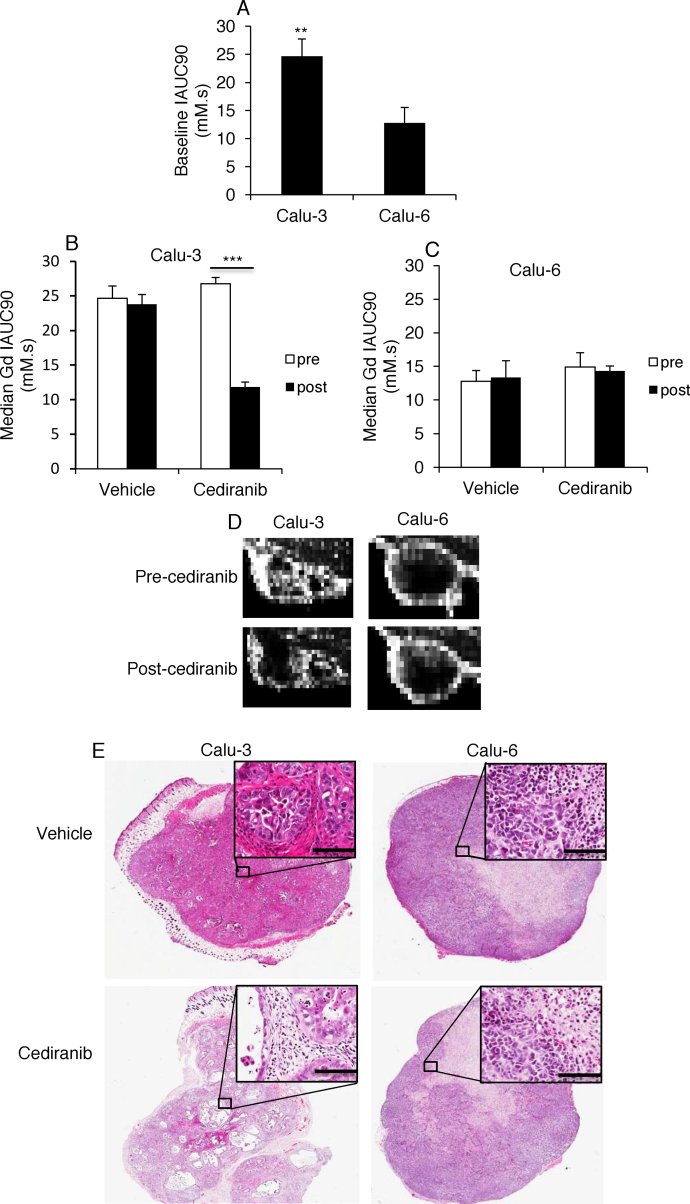
Acute vascular response to cediranib treatment in NSCLC xenografts. Mice bearing Calu-3 or Calu-6 tumours were treated with cediranib (6 mg/kg) or vehicle at 0 h and 22 h. DCE-MRI imaging was performed 2 h before the first and 2 h after the second treatment. (A) DCE-MRI analysis of basal tumour perfusion. Calu-3 tumours show significantly higher gadolinium uptake compared to Calu-6 tumours. **P < 0.01 (B) The average IAUC values of Calu-3 xenografts pre- and post-treatment. Compared to the baseline, gadolinium uptake was significantly reduced in Calu-3 tumours by cediranib treatment. ***P < 0.001 (C) The average IAUC values of Calu-6 xenografts pre- and post-treatment. Compared to the baseline, gadolinium uptake was not affected by cediranib in Calu-6 tumours. (D) Representative MRI images from single slides of Calu-3 and Calu-6 tumours pre- and post-cediranib treatment. (E) Tumour H&E histological staining in vehicle- or cediranib- treated Calu-3 and Calu-6 tumours. Acellular regions were widespread in cediranib-treated Calu-3 tumours, but were rare in vehicle-treated Calu-3 tumours. No difference in necrotic fraction between vehicle- and cediranib-treated Calu-6 tumours was noted. Scale bars = 100 μm.

DCE-MRI measures both perfusion and permeability. Thus, to evaluate the impact of cediranib on tumour vascular function, animals bearing Calu-3 or Calu-6 xenografts were treated with cediranib or vehicle twice, 22 h apart. DCE-MRI was performed 2 h before the first dose and 2 h after the second dose. The time points chosen for DCE-MRI were based on the fact that cediranib has a half-life of 22 h [25], and reaches its maximum effect at 2 h in mice after a single oral dosing [26]. The median IAUC was calculated for the tumour in each animal. The average baseline IAUC of Calu-3 tumours was significantly higher than that of Calu-6 tumours (*P *< 0.01) (Fig. 2A), suggesting that Calu-3 tumours were better-perfused than Calu-6 tumours. The median IAUC in Calu-3 tumours was reduced by 55.85 ± 4.06% after cediranib administration (pre- vs post-cediranib: *P *< 0.001), whereas IAUC in Calu-3 tumours was not significantly affected by vehicle treatment (*P *= 0.727) (Fig. 2B). In contrast, the median IAUC of Calu-6 tumours was not significantly changed by vehicle or cediranib treatment (pre- vs post-vehicle: *P *= 0.857; pre- vs post-cediranib: *P *= 0.803) (Fig. 2C). Calu-3 tumours at baseline displayed uniform and high perfusion, but gadolinium uptake was sharply reduced after cediranib treatment (Fig. 2D). Calu-6 tumours at baseline showed a well-perfused rim with a poorly perfused central region, which was unchanged after cediranib treatment (Fig. 2D). Taken together, these data suggest that Calu-3 tumour vessels are more sensitive to cediranib treatment.

To relate changes in IAUC to histopathology, the extent of necrosis was assessed by H&E staining (Fig. 2E). Necrosis was not evident in vehicle-treated Calu-3 tumours, but cediranib treatment resulted in widespread acellular regions throughout the tumours, consistent with the hypointense regions seen in Calu-3 DCE-MRI images. In contrast, a necrotic core was evident in both vehicle- and cediranib-treated Calu-6 tumours, corresponding to the hypointense central region seen in Calu-6 DCE-MRI images. There was no evident difference in necrotic fraction between vehicle-treated and cediranib-treated Calu-6 tumours.

### 3.3 Acute hypoxia is induced in Calu-3 tumours following cediranib

**Fig. 3:**
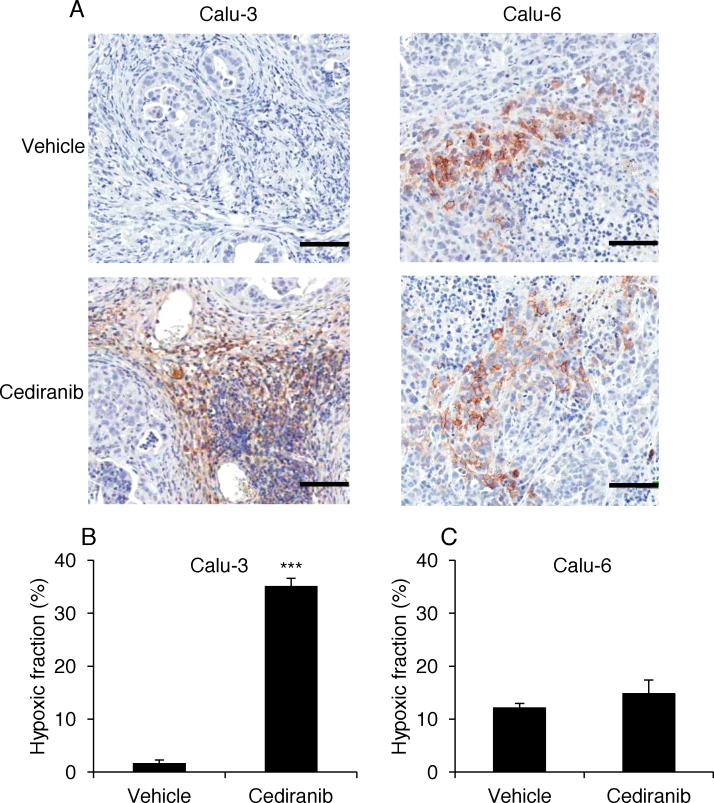
Immunohistochemical detection of tumour hypoxia. Tumour-bearing mice treated with two doses of cediranib or vehicle were sacrificed after DCE-MRI. Tumours were sectioned and hypoxia was identified by CA9 immunohistochemistry. (A) CA9 staining in vehicle or cediranib treated Calu-3 and Calu-6 tumours. Vehicle-treated Calu-3 tumours showed rare CA9 staining, whereas cediranib-treated Calu-3 tumours showed extensive CA9 positive areas. Calu-6 tumours showed similar levels of peri-necrotic staining of CA9 in both vehicle- and cediranib-treated animals. Scale bars = 100 μm. (B) Hypoxic fractions in Calu-3 tumours treated with vehicle or cediranib. ***P < 0.001. (C) Hypoxic fractions in Calu-6 tumours treated with vehicle or cediranib.

To investigate whether the tumour perfusion change resulted in oxygenation changes, tumour hypoxia was assessed by immunohistochemical staining for the endogenous hypoxia marker CA9 [27], in Calu-3 and Calu-6 tumours harvested 2 h after the second dose of cediranib or vehicle treatment. CA9 positive staining was rare in vehicle-treated Calu-3 tumours (Fig. 3A and B), suggesting that Calu-3 tumours are well-oxygenated, whereas cediranib-treated Calu-3 tumours showed significantly higher hypoxic fractions compared to the vehicle-treated control (*P *< 0.001; Fig. 3A and B), suggesting that cediranib treatment induced acute hypoxia, consistent with reduced perfusion. In comparison, CA9 staining in Calu-6 tumours was evident in perinecrotic areas of the tumours (Fig. 3A) and hypoxic fractions in cediranib- and vehicle-treated animals were similar (14.8 ± 2.6% vs 12.1 ± 0.9%; *P *= 0.382; Fig. 3C), suggesting no significant effect of cediranib on Calu-6 tumour oxygenation.

### 3.4 Reduction in tumour perfusion associates with tumour growth inhibition

**Fig. 4:**
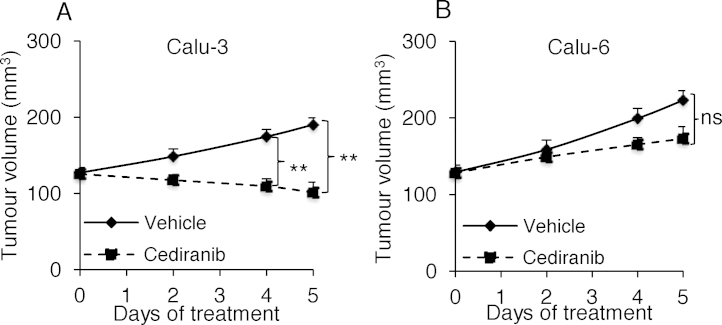
Effects of cediranib on tumour growth in Calu-3 and Calu-6 xenograft models. Calu-3 or Calu-6 tumour-bearing mice were treated with either cediranib or vehicle by oral gavage once daily for 5 days. Tumour volumes were measured and growth curves were plotted. (A) Calu-3 tumour growth curves. Cediranib treated Calu-3 tumours showed a significantly smaller tumour size and tumour regression compared to vehicle-treated controls. **P < 0.01. (B) Calu-6 tumour growth curves. Cediranib treated Calu-6 tumours showed a trend towards growth inhibition, but this did not reach statistical significance. ns: not significant.

To evaluate whether the early reduction in tumour perfusion was associated with tumour growth inhibition, mice bearing Calu-3 and Calu-6 tumours were treated with cediranib once daily for 5 days, and tumour volumes were measured and plotted. Regression of Calu-3 tumours was evident as early as 2 days after the onset of cediranib treatment, and persisted throughout the whole treatment course. Statistically significant growth inhibition by cediranib in Calu-3 tumours was obtained on day 4 post-treatment, and continued till the experiment's end on day 5. (*P *< 0.01) (Fig. 4A). However, no shrinkage of Calu-6 tumours was observed. Although there was a trend for cediranib to inhibit Calu-6 tumour growth after 5 days cediranib treatment (Fig. 4B), it did not reach statistical significance (*P* = 0.07).

## 4 Discussion

It has been suggested that the vascular phenotypes of tumours categorised by stromal architecture can define tumour response to chronic VEGF-targeted monotherapy [14]. However, the impact of this phenotypic variability on acute tumour vascular response to VEGFR TKI treatment has not been reported. To extend the previous study [14], we investigated the acute vascular response to cediranib in two NSCLC xenograft models representing tumour vessel and stromal vessel phenotypes, respectively, and compared the acute vascular responses with tumour growth inhibition. We found that the perfusion of Calu-3 tumours (stromal vessel phenotype) was rapidly reduced after cediranib administration, leading to acute hypoxia (within 24 h). In comparison, neither the perfusion nor hypoxia was significantly affected by cediranib in Calu-6 tumours (tumour vessel phenotype). Moreover, tumour regression was induced in Calu-3 xenografts, but not in Calu-6 xenografts after 5 days of cediranib treatment, although there was a trend towards tumour growth inhibition in Calu-6 xenografts. Taken together, our results suggest that tumour stromal architecture may be associated with acute tumour vascular response to VEGFR TKI.

Previous gene expression analysis has shown that the two vascular phenotypes of tumours exhibit differential gene expression profiles [14]. Compared with the tumour vessel phenotype, tumours with stromal vessels express higher levels of certain genes associated with recruitment of stromal cells, most notably PDGF and FGF [14]. In addition, vasculature of the tumour vessel phenotype tends to be pericyte-free, whereas vasculature of the stromal vessel phenotype is pericyte covered [14]. Vasculature with pericyte coverage is less sensitive to VEGF inhibition, but anti-VEGF/VEGFR therapy may be improved by concomitant inhibition of PDGFR expressed on pericytes [28]. Hence, cediranib treatment by inhibiting pan-VEGFR, PDGFR, and FGFR may have a greater effect on tumours with a stromal vessel phenotype than treatment with a highly selective VEGFR2-signalling inhibitor such as DC101.

Acute vascular response is characteristic of vascular disrupting agents (VDAs) [29]. Our data here suggest that cediranib may be acting, at least in part, as a VDA on the stromal vasculature in Calu-3 xenografts. However, the VEGFR2 blocking antibody, DC101 did not show significant effect in the Calu-3 xenograft model [14], suggesting inhibition of other VEGF receptors, PDGFR and FGFR signalling may be required to acutely disrupt vessel function in this model.

In contrast, no significant effect of acute cediranib treatment was detected in Calu-6 tumours by using DCE-MRI. It has previously been reported that cediranib-induced vessel pruning was only apparent in the periphery of the Calu-6 tumours [26]. Since vessel pruning occurs preferentially in immature or non-functional blood vessels [30], the overall impact of cediranib treatment on established functional tumour vasculature may be limited in Calu-6 xenografts and therefore could not be detected by DCE-MRI. In another DCE-MRI study [31], cediranib treatment reduced *K*^trans^, a biomarker of tumour perfusion and vessel permeability, after 3 or 5 days of treatment in Calu-6 xenografts, suggesting that more prolonged treatment with cediranib can produce significant anti-vascular effects. Nonetheless, in agreement with our study, no tumour growth inhibition was observed over 5 days of cediranib treatment. This is consistent with other findings whereby significant tumour growth inhibition was only evident in Calu-6 xenografts after 2 weeks of DC101 treatment [14]. These results suggest that the response of Calu-6 xenografts to cediranib treatment is characteristic of VEGF signalling inhibition in human tumour xenografts, whereby more prolonged treatment is required [32], which is associated with changes in vessel permeability, increased vascular normalisation [33] and inhibition of new blood vessel development [34].

Although xenograft models in NSCLC and other cancers exhibit distinct vessel phenotypes, human NSCLC tumours are histologically heterogeneous as shown in our study: a high proportion of tumours having a mixed vessel phenotype, with both tumour vessel and stromal vessel phenotypes present within the same tumour. In addition, in our analysis, only 1/38 human NSCLC tumour samples had a predominantly tumour vessel phenotype compared with 10/12 NSCLC xenograft models. This suggests that the tumour vasculature in xenograft models is not a good representation of the clinical situation. Therefore, defining patient subgroups by tumour stromal architecture can be difficult in the clinic, especially where only small biopsies are available.

Of interest, Calu-3 tumours were better and more uniformly perfused throughout the tumour than the Calu-6 xenografts which were well-perfused only at the tumour rim, suggesting that DCE-MRI may provide a potential non-invasive biomarker to discriminate stromal and tumour vessel phenotypes. It has been reported that acute changes in tumour vessel function are more predictive of tumour growth response than changes in histology or gene profiles [35]. Moreover, quantitative DCE-MRI has be used in breast cancer xenografts as an early predictor of response to bevacizumab treatment [36] and neoadjuvant chemotherapy [37], [38]. Comparable to those findings, our study showed that reduced tumour perfusion following cediranib was associated with enhanced tumour growth inhibition, indicating that acute reduction in tumour perfusion could be an early predictive marker of response to vascular targeted therapies in NSCLC. Indeed, some evidence suggests that reduction in tumour perfusion associates with better response to VEGF-inhibitor therapy in lung cancer. After bevacizumab treatment, NSCLC patients with more than 20% reduction in tumour perfusion had a longer progression-free survival [39]. Another clinical study on NSCLC patients treated with anti-angiogenic chemotherapy also showed that tumour vascular volume was reduced significantly in responders versus non-responders [40]. In addition, decreased tumour blood volume during antiangiogenic therapy was associated with a clinic benefit in lung cancer patients [41].

It is noteworthy that reduced perfusion can lead to acute hypoxia as shown in Calu-3 tumours. Increased hypoxia might decrease the efficacy of cytotoxic therapies when anti-VEGF/VEGFR treatment is combined with radiotherapy or chemotherapy. However, some studies have shown that even in the case of tumour hypoxia induced by antiangiogenic therapy, the antitumour effects can still be enhanced by combined chemotherapy [42], [43]. The underlying mechanism might be independent of hypoxia. Future studies are warranted to explore the effects of cediranib in combination with chemo- or radiotherapy in lung cancer.

In this study, acute vascular response to cediranib was evaluated in two NSCLC xenograft models. Future studies are warranted to evaluate a broad range of xenograft models in lung cancer and other tumour types. Furthermore, it will be of interest to determine the correlation between the acute vascular response to VEGFR TKI and the long-term outcome in NSCLC patients.

In conclusion, we propose that tumour stromal architecture may affect the response of tumour vasculature to VEGFR TKI treatment, and that acute change in tumour perfusion may be predictive of tumour response to VEGFR TKI in NSCLC.

## Conflict of interest

None declared.
